# Preparation of Artificial Plasma Membrane Mimicking Vesicles with Lipid Asymmetry

**DOI:** 10.1371/journal.pone.0087903

**Published:** 2014-01-28

**Authors:** Qingqing Lin, Erwin London

**Affiliations:** Department of Biochemistry and Cell Biology, Stony Brook University, Stony Brook, New York, United States of America; Cornell University, United States of America

## Abstract

Lipid asymmetry, the difference in lipid distribution across the lipid bilayer, is one of the most important features of eukaryotic cellular membranes. However, commonly used model membrane vesicles cannot provide control of lipid distribution between inner and outer leaflets. We recently developed methods to prepare asymmetric model membrane vesicles, but facile incorporation of a highly controlled level of cholesterol was not possible. In this study, using hydroxypropyl-α-cyclodextrin based lipid exchange, a simple method was devised to prepare large unilamellar model membrane vesicles that closely resemble mammalian plasma membranes in terms of their lipid composition and asymmetry (sphingomyelin (SM) and/or phosphatidylcholine (PC) outside/phosphatidylethanolamine (PE) and phosphatidylserine (PS) inside), and in which cholesterol content can be readily varied between 0 and 50 mol%. We call these model membranes “artificial plasma membrane mimicking” (“PMm”) vesicles. Asymmetry was confirmed by both chemical labeling and measurement of the amount of externally-exposed anionic lipid. These vesicles should be superior and more realistic model membranes for studies of lipid-lipid and lipid-protein interaction in a lipid environment that resembles that of mammalian plasma membranes.

## Introduction

A common feature of eukaryotic cell membranes is the non-random distribution of lipid species in the inner and outer leaflets of the lipid bilayer, which is called lipid asymmetry. In mammalian plasma membranes aminophospholipids (phosphatidylserine (PS) and phosphatidylethanolamine (PE)) are predominantly exposed on the cytosolic leaflet, whereas phosphatidylcholine (PC) and sphingolipids, including sphingomyelin (SM), are predominantly located on the outer leaflet [1], [2]. This transmembrane (TM) lipid asymmetry provides the two sides of plasma membrane with different biophysical properties and influences numerous cellular functions. For instance, cytoplasmic-located anionic phospholipid PS is an essential co-factor for a number of membrane-bound enzymes, such as protein kinase C and Na^+^/K^+^-ATPase [3], while externalization of PS on the cell surface acts as a recognition site for phagocytes, and promotes the blood coagulation cascade [4]. Lipid asymmetry also imparts asymmetry of lipid charge across the bilayer, with a higher anionic charge at the cytofacial surface of membranes. This may influence membrane protein topology and the establishment of TM protein orientation during biosynthesis [5].

Thus, lipid asymmetry is an important membrane property that merits detailed study. Model membrane vesicles, which avoid the complexity of natural membranes, should be an ideal system for studies of the effects of lipid structure upon membrane and protein properties. However, although they have contributed much to our understanding of biological membranes and lipid-protein interaction, the inability to prepare model membrane vesicles with highly controlled lipid asymmetry has limited their value in this regard. Some progress has been made in preparing asymmetric model membrane vesicles, such as by inserting a specific lipid to pre-formed liposomes, either spontaneously [6], [7] or by using pH gradients, or by the exchange of short-chain lipids between liposome populations [8]. However, the level of lipid exchange and the degree of asymmetry control has been limited. More recently, our group introduced a method using lipid exchange induced by methyl-β-cyclodextrin (MβCD) to carry out efficient and highly controlled exchange with diverse lipids [9], [10]. The method has been applied to small, large and giant unilamellar vesicles [9], [11], [12].

However, one important limitation of the MβCD-based exchange method has been the lack of a simple method to easily incorporate a range of highly controlled levels of cholesterol. The method developed in our previous studies required a second step of lipid exchange that resulted in low vesicle yields and poor control of cholesterol levels. In the present study, the cyclodextrin-induced exchange method was extended to use of (2-hydroxylpropyl)-α-cyclodextrin (HPαCD) in order to prepare asymmetric large unilamellar vesicles (LUV) containing a wide range of highly controlled amounts of cholesterol. HPαCD has a smaller ring size than MβCD and shows no/little affinity for cholesterol, but we recently found it retains the ability to mediate phospholipid exchange [13]. Using this method, asymmetric vesicles were prepared with an outer leaflet rich in SM, or PC, or a mixture of SM and PC and an inner leaflet rich in PE and PS and with various amounts of cholesterol (from 0–50 mol%). Thus, vesicles that closely mimic natural plasma membrane of mammalian cells can now be prepared.

## Materials and Methods

### Materials

Porcine brain sphingomyelin (SM); 1-palmitoyl-2-oleoyl-phosphatidylcholine (POPC); 1-palmitoyl-2-oleoyl-phosphatidylethanolamine (POPE); 1-palmitoyl-2-oleoyl-L-serine (POPS); cholesterol (CHOL) and 1-palmitoyl-2-6-[(7-nitro-2-1,3-benzoxadiazol-4-yl)amino]hexanoyl- phosphatidylcholine (C_6_-NBD-PC) were purchased from Avanti Polar Lipids (Alabaster, AL). [^3^H]-cholesterol was purchased from American Radiolabeled Chemicals, Inc (St. Louis, MO). Lipids were dissolved in chloroform and stored at −20°C. Concentrations of lipids were measured by dry weight. (2-hydroxypropyl)-α-cyclodextrin (HPαCD) average molecular weight 1180, 1 M 2,4,6-trinitrobenzenesulfonic acid in water (TNBS) and sodium hydrosulfite (sodium dithionite) were purchased from Sigma-Aldrich (St. Louis, MO). LW peptide (acetyl-K_2_W_2_L_8_AL_8_W_2_K_2_-amide) and pL4A18 peptide (acetyl-K_2_LA_9_LWLA_9_LK_2_-amide) were purchased from Anaspec (San Jose, CA) and used without further purification. High-performance thin-layer chromatography (HP-TLC) plates (Silica Gel 60) were purchased from VWR International (Batavia, IL).

### Ordinary (symmetric) vesicle preparation

To prepare multilamellar vesicles (MLV), lipid mixtures were mixed and dried under nitrogen followed by high vacuum for at least 1 h. The dried lipid mixtures were then dispersed in phosphate-buffered saline (PBS, 1.8 mM KH_2_PO_4_, 10 mM Na_2_HPO_4_, 137 mM NaCl, and 2.7 mM KCl at pH 7.4), at 70°C to give the desired final concentration and agitated at 55°C for 15 min using a VWR multitube vortexer (Westchester, PA) placed within a convection oven (GCA Corp, Precision Scientific, Chicago, IL). Samples were cooled to room temperature before use.

Large unilamellar vesicles (LUV) were prepared from MLV by subjecting the MLV to 7 cycles of freezing in a mixture of dry ice and acetone and thawing at room temperature and then passed through 100-nm polycarbonate filters (Avanti Polar Lipids) 11 times to obtain LUV of uniform vesicle size. In the case of “acceptor” LUV used to prepare asymmetric vesicles (see below), lipids were dispersed in a solution containing 25% (w/v) sucrose dissolved in water instead of PBS. To wash away the untrapped sucrose, the resulting LUV solutions (500 µl) were mixed with 3.5 ml PBS and subjected to ultracentrifugation at 190,000 g for 30 min using Beckman L8-55 M ultracentrifuge and a SW 60 rotor. After the supernatant was discarded, the LUV pellet was resuspended with PBS to the desired concentration (unless otherwise noted, 8 mM, assuming no loss during centrifugation) for further experiments.

### Asymmetric LUV preparation

Asymmetric large unilamellar vesicles were formed using a protocol adapted from that described by Cheng *et al.* [11]. First, 8 µmol SM or POPC or SM/POPC (“donor”) mixtures in chloroform were dried under nitrogen and high vacuum for at least 1 h. Then the dried lipids were hydrated with 150 µl of 420 mM HPαCD at 70°C and diluted with 450 µl PBS. This donor mixture was vortexed in the multitube vortexer at 55°C overnight. The next day, the mixture was sonicated in a bath sonicator (Special Ultrasonic Cleaner Model G1112SP1, Laboratory Supplies Co., Hicksville, NY) at room temperature for 15 min before adding 500 µl of 8 mM (total lipid) acceptor POPE/POPS/cholesterol LUVs (with entrapped 25% (w/v) sucrose; see above). The amount of cholesterol used in the acceptor vesicles was varied between 0 and 50 mol% depending on the specific experiment. The mixture of donor POPC/HPαCD and acceptor POPE/POPS/cholesterol LUVs was vortexed at 55°C for 30 min, overlaid onto 3 ml of a 10% (w/v) sucrose solution, and then subjected to ultracentrifugation at 190,000 g for 30 min at a setting of 25°C. After the supernatant was removed, the resulting pellet was resuspended with 1 ml of 10% (w/v) sucrose solution, overlaid onto 3 ml of a 10% (w/v) sucrose solution, and the ultracentrifugation step was repeated. The final pellet was resuspended in PBS and immediately used for further experiments. Generally the lipid yield from this preparation was ∼1 µmol as measured by the intensity of lipid bands on HP-TLC relative to lipid standards (see below). In cases in which lipid concentration was not explicitly measured, 1 µmol was assumed as the lipid yield per preparation unless otherwise noted.

### High performance thin layer chromatography

Asymmetric LUV samples were extracted using 2∶2∶1 (v/v) chloroform/methanol/aqueous sample. After 5 min of low-speed centrifugation, the upper aqueous phase was discarded and the lower phase (containing the lipid extract) was dried under nitrogen. The extracted lipids or pure lipid standards were applied to HP-TLC (Silica Gel 60) plates and chromatographed using a dual solvent system as described previously [9]. The band intensity on the charred HP-TLC plates [9] was measured by Un-Scan-It software (Silk Scientific Inc., Orem, UT). Lipids in samples were quantified by comparing band intensity with that of the standards fit to an exponential intensity versus concentration curve (SlideWrite Plus Software, Rancho Santa Fe, CA).

### Dynamic light scattering

The size of LUV was measured by Protein Solutions DynaPro 99 dynamic light-scattering instrument (Wyatt Technology, Santa Barbara, CA) at 23°C. The size of LUV before and after exchange was determined by diluting the vesicles 500- to 1000-fold with 0.22-µm-filtered PBS. Data were analyzed with the Dynamics V5.25.44 program supplied by Wyatt Technology.

### Fluorescence Intensity Measurements

Fluorescence emission intensity was measured (unless otherwise noted) at room temperature on a SPEX Fluorolog 3 spectrofluorimeter. For fixed wavelength measurements, excitation, emission wavelength sets used (in nm) were (280, 340) for tryptophan and (465, 534) for C_6_-NBD-PC. For pL4A18 peptide tryptophan emission spectra measurements, samples were excited at 280 nm and emission spectra were acquired from 325–380 nm. Unless otherwise noted, fluorescence intensity in single background samples lacking fluorophore was subtracted from the reported data.

### Sucrose density gradient centrifugation

Sucrose gradient centrifugation was carried out similarly to as described by Cheng *et al.* [9]. Gradients were prepared by freeze-thawing 3.4 ml of 20% (w/v) sucrose overnight at −20°C in the centrifuge tubes (Beckman ultraclear). 400 µl of vesicle samples (∼0.4 µmol lipid) were loaded on top of the gradients and centrifuged at 190,000 g, at 4°C for 17 h. After centrifugation the gradients were fractioned by pipetting into 300 µl aliquots, starting at the top (The bottom, the highest density fraction contained 200–400 µl). Lipids from each fraction were then extracted and applied to HP-TLC as described above.

### Measurement of contamination from “donor” vesicles

“Donor” mixtures composed of POPC and HPαCD were prepared as described above except containing 0.1 mol% C_6_-NBD-PC. After vortexing at 55°C overnight and sonication for 15 min, 80 µl of 1 M sodium dithionite freshly prepared in 1 M Tris pH 10 was added to quench the NBD fluorescence from C_6_-NBD-PC located in the outer leaflet of the residual POPC MLV and in C_6_-NBD-PC/HPαCD complexes. Acceptor POPE/POPS/40 mol% cholesterol LUVs (with entrapped 25% (w/v) sucrose) prepared as described above were then added and vortexed at 55°C for 30 min. After cooling to room temperature the NBD emission intensity was measured after vortexing. After two rounds of ultracentrifugation on 10% (w/v) sucrose (as above), the NBD emission intensity was re-measured at room temperature for the supernatant from each round of centrifugation and from the final pellet. The amount of POPC in the supernatants and pellet was also measured by HP-TLC, as described above.

### Measurement of cholesterol extraction by HPαCD in symmetric LUV

Cholesterol extraction from vesicles by HPαCD was assessed by ultracentrifugation. 100 µl of 6∶4 POPC/cholesterol LUVs (10 mM lipid) containing 0.5 mol% LW-peptide and 2.5 µCi [^3^H]-cholesterol were prepared in 25% (w/v) sucrose solution. After diluting with 860 µl PBS and adding 40 µl of 420 mM HPαCD, samples were vortexed at 55°C for 30 min as above and then subjected to ultracentrifugation at 84,000 g at 4°C for 30 min. After centrifugation, supernatant containing HPαCD, and HPαCD bound lipid, was removed and the pellet containing the LUVs was resuspended with 1 ml PBS pH 7.4. Then Trp fluorescence intensity and [^3^H]-cholesterol radioactivity in both supernatant and pellet was measured. The radioactivity to Trp ratio was used to calculate the mol% content of cholesterol in the LUVs after HPαCD treatment.

### Measurement of cholesterol extraction by HPαCD in asymmetric LUVs preparations

Asymmetric LUVs composed of POPC outside and POPE/POPS/cholesterol inside were prepared as described earlier, except that 0.2 mol% LW-peptide and 10 µCi [^3^H]-cholesterol were incorporated into the acceptor vesicles before lipid exchange. Trp fluorescence intensity and [^3^H]-cholesterol radioactivity were measured for the two supernatants and the final pellet from two rounds of centrifugation.

### Peptide-vesicle interaction

To examine the interaction between pL4A18 peptide and vesicle, 1 mol% (relative to lipid) of pL4A18 peptide was added to 1 ml of symmetric or asymmetric LUVs containing 100 µM lipids in PBS pH 7.4. After 10 min incubation at room temperature Trp emission spectra were measured.

### TNBS-labeling

To test the labeling of aminophospholipids on the outer leaflet of symmetric LUVs, 900 µl of 1 mM TNBS freshly prepared in 100 mM NaHCO_3_ pH 9 was added to 100 µl symmetric LUVs (2 mM lipid) in PBS pH 7.4 and incubated at room temperature for various times. The reaction was terminated by addition of 200 µl, concentrated HCl and lipids were extracted with 4 ml 1∶1 (v/v) methanol/chloroform and applied on HP-TLC.

To label the aminophospholipids on both leaflets of symmetric LUVs, 100 µl symmetric LUVs (2 mM lipid) in PBS pH 5.1 were incubated with ∼20 µg pore-forming protein perfringolysin O (PFO) [14], for 1 h and then diluted with 900 µl PBS pH 5.1. The PFO-bound vesicles were spun down for 30 min at 84,000 g at 4°C. The pellets were resuspended with 100 µl PBS pH 7.4 and mixed with 900 µl of 1 mM TNBS freshly prepared in 100 mM NaHCO_3_ pH 9 for various times at room temperature. Reaction termination, lipid extraction and analysis of HP-TLC were the same as described above.

To label the outer leaflet of asymmetric LUVs, 100 µl asymmetric LUVs containing ∼0.2 µmol lipid in PBS pH 7.4 were incubated with 900 µl of 1 mM TNBS freshly prepared in 100 mM NaHCO_3_ pH 9 for 1 h at room temperature. Reaction termination, lipid extraction and analysis of HP-TLC were the same as described above.

## Results

### Preparation of cholesterol-containing asymmetric (exchange) LUVs

Our first aim was to define methods to prepare asymmetric vesicles with various controlled amounts of cholesterol, and with a composition that would roughly approximate that of mammalian plasma membranes. This requires defining conditions for efficient lipid exchange, ruling out artifacts that mimic lipid exchange (e.g. vesicle aggregation), and then assaying the degree of lipid asymmetry in the resulting vesicles. The strategy chosen was to prepare vesicles with the desired amounts of cholesterol and then carry out cyclodextrin-catalyzed lipid exchange under conditions in which cholesterol would not exchange. To do this we used HPαCD, which has a smaller ring size than the MβCD used previously [15], [16]. Cholesterol should not fit well into the cavity of HPαCD, and thus should not be subject to exchange, but fatty acids should fit, and so phospholipid exchange should be possible [13], [17].

**Figure 1:**
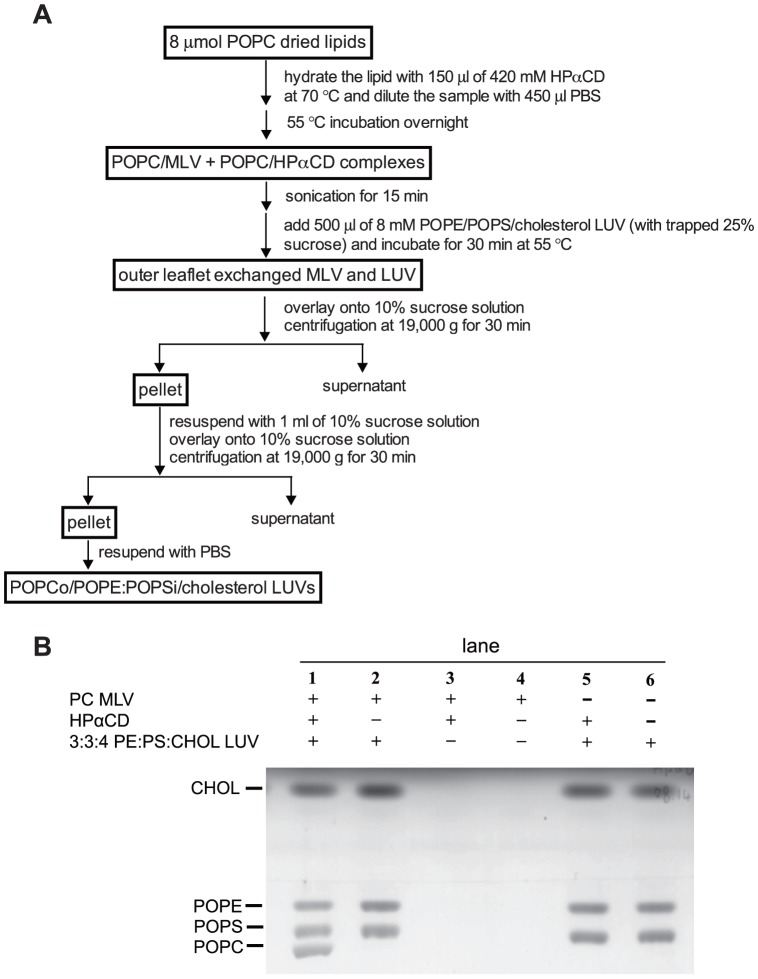
Preparation of asymmetric LUVs. (A) Flow chart of method for preparation of asymmetric POPCo/POPE:POPSi/cholesterol LUVs. Preparation of vesicles with SM or SM/POPC outside is identical to POPC except that the initial dried lipids are different. The concentration of donor lipids and HPαCD after overnight incubation at 55°C is slightly higher (by ∼10–20%) than theoretical because of evaporation. (B) HP-TLC analysis of final pellet from asymmetric LUVs protocol. POPC MLVs (lane 1 to 4) or PBS (lane 5 and 6) were incubated with (lane 1, 3 and 5) or without (lane 2, 4, and 6) HPαCD overnight and 3∶3∶4 POPE/POPS/cholesterol LUVs (lanes 1, 2, 5 and 6) or PBS (lanes 3 and 4) were then added, and incubated for 30 min at 55°C, followed by two centrifugation steps.

Cholesterol-containing asymmetric LUVs with POPC, SM or a SM/POPC mixture in the outer leaflet and POPE/POPS in the inner leaflet were prepared by HPαCD-induced lipid exchange, and the lipid composition of the resulting vesicles assayed by HP-TLC. Fig. 1A shows a schematic summary of the exchange procedure. The lipids forming the outer leaflet of the asymmetric vesicles come from donor MLVs, and the lipids forming the inner leaflet are in acceptor LUVs. Ultracentrifugation is used to separate donor vesicles (POPC in most studies) from exchanged acceptor vesicles (POPE/POPS/cholesterol) which contain 25% (w/v) sucrose trapped inside to induce pelleting. POPC MLVs (which do not have trapped sucrose) have a lower density and float after centrifugation.

As shown by HP-TLC in Fig. 1B (lane 1), when donor vesicles contained POPC, after exchange, the pelleted vesicles (“exchange vesicles”) contained POPC as well as POPE, POPS and cholesterol. The appearance of POPC in the pelleted vesicles was dependent upon the presence of HPαCD (lane 2). POPC donor vesicles did not pellet in the absence of acceptor vesicles both with (lane 3) and without (lane 4) HPαCD present, whereas the acceptor POPE/POPS/cholesterol vesicles pelleted in the absence of donor vesicles both with (lane 5) and without (lane 6) HPαCD present.

As judged by visual inspection of cholesterol band intensity, Fig. 1B also suggests that the level of cholesterol in pelleted (acceptor or exchange) vesicles was not significantly reduced in the presence of HPαCD and/or donor vesicles. Ultracentrifugation experiments using [^3^H]-cholesterol, and the TM LW peptide as a non-exchangeable marker [9], confirmed that cholesterol was not extracted from acceptor vesicles by HPαCD (Fig. S1 in File S1).

We also investigated whether the donor lipid/acceptor lipid ratio would affect the efficiency of exchange. Because we found that increasing donor vesicle or HPαCD concentration degrades vesicle separation during ultracentrifugation, acceptor vesicle concentrations were varied while donor vesicle concentration and HPαCD concentration remained fixed. HP-TLC analysis showed that higher donor/acceptor lipid ratios gave a higher amount of lipid exchange into acceptor vesicles (Fig. S2 in File S1), but resulted in a lower yield of exchange vesicles (not shown). Therefore, we chose a 2∶1 mol:mol donor/acceptor ratio to prepare asymmetric vesicles in the following studies. (All ratios given below are also mol:mol.)

### Association of donor and acceptor lipids reflects lipid exchange

**Figure 2:**
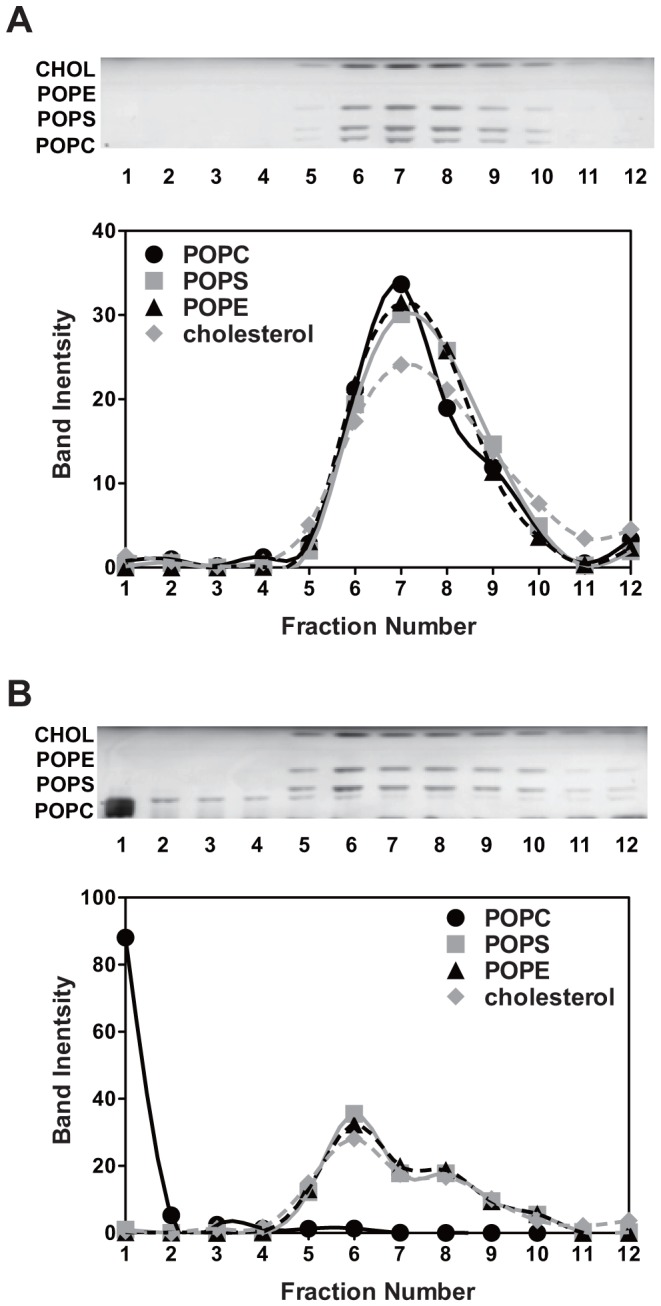
Sucrose density gradient centrifugation of POPCo/POPE:POPSi/cholesterol vesicles. (A) Sucrose gradient fractions of exchange LUVs containing ∼400 µl of 1 mM (total lipid) POPCo/POPE:POPSi/40 mol% cholesterol. (B) Sucrose gradient fractions of a mixture of 200 µl of 1 mM POPC MLVs with HPαCD and 200 µl of 1 mM LUVs composed of 3∶3∶4 POPE/POPS/cholesterol with 25% (w/v) entrapped sucrose. Top: stained TLC plate; Bottom: Band intensity for each lipid. Fractions from left to right are from low to high density. Gradients were prepared by freeze-thawing 3.4 ml of 20% (w/v) sucrose. 400 µl of vesicle samples were loaded on top of the gradients and the gradients were centrifuged for 17 h at 19,000 g.

Association of donor and acceptor lipids might not reflect lipid exchange. To rule out the possibility that the presence of POPC in the pellet after the lipid exchange step was due to sticking of POPC vesicles to POPE/POPS/cholesterol LUVs rather than true lipid exchange, sucrose density gradient centrifugation was used. Fig. 2A shows the HP-TLC analysis of different density fractions for POPCo/POPE:POPSi/cholesterol exchange vesicles with entrapped 25% (w/v) sucrose. (Exchange vesicle names have the format Xo/Yi/cholesterol, where Xo designates the original donor lipid(s), which should be in the outer leaflet and Yi designates the original acceptor vesicle lipid(s), which should be in the inner leaflet.) As expected, all lipid species co-fractionated in the medium density fractions. In contrast, when the POPC/HPαCD mixture and POPE/POPS/cholesterol LUVs (with 25% (w/v) sucrose trapped) were mixed at room temperature and immediately loaded onto gradients without the 30 min, 55°C exchange step, POPC located in the lowest density fractions, whereas the POPE/POPS/cholesterol vesicles located in the medium density fractions (Fig. 2B). A similar result was observed in the absence of HPαCD (not shown). This means POPC vesicles do not stick to POPE/POPS/cholesterol vesicles, and thus vesicle sticking does not explain co-pelleting of POPC with POPE/POPS/cholesterol after exchange.

Another concern was that POPC and POPE/POPS/cholesterol co-pellet due to vesicle fusion. This was ruled out by the observation that acceptor LUV size (radius 65–70 nm) remained unchanged or increased minimally after lipid exchange (Fig. S3 in File S1).

To fully rule out the possibility of substantial contamination of exchange vesicles by donor vesicles we carried out exchange between POPC donor vesicles labeled with the fluorescent lipid C_6_-NBD-PC and POPE/POPS/cholesterol acceptor vesicles. Immediately prior to lipid exchange C_6_-NBD-PC in the POPC MLVs outer leaflet and in C_6_-NBD-PC loaded HPαCD was destroyed by sodium dithionite reduction, to form the non-fluorescent C_6_-ABD-PC derivative [18]. C_6_-NBD-PC in the inner leaflet and in internal vesicles of the POPC MLVs will be protected from reduction by dithionite added to the external solution. Because exchange involves the exchange of lipids in donor lipid/HPαCD complexes and in donor vesicle outer leaflets with acceptor vesicle lipids, fluorescent C_6_-NBD-PC should not be involved in lipid transfer. Thus, after centrifugation NBD fluorescence in the pellet is a sign of contamination from donor vesicles (see protocol summary in Fig. S4 in File S1). Table S1 in File S1 shows that based on this assay only 2.5% (or less if dithionite reduction is incomplete) of the lipid in the pellet containing exchange vesicles came from contaminating donor vesicles.

In summary, the appearance of POPC in the pelleted vesicles is almost all due to lipid exchange.

### Preparation and lipid composition of asymmetric LUV containing different amounts of cholesterol, SM and POPC

**Figure 3:**
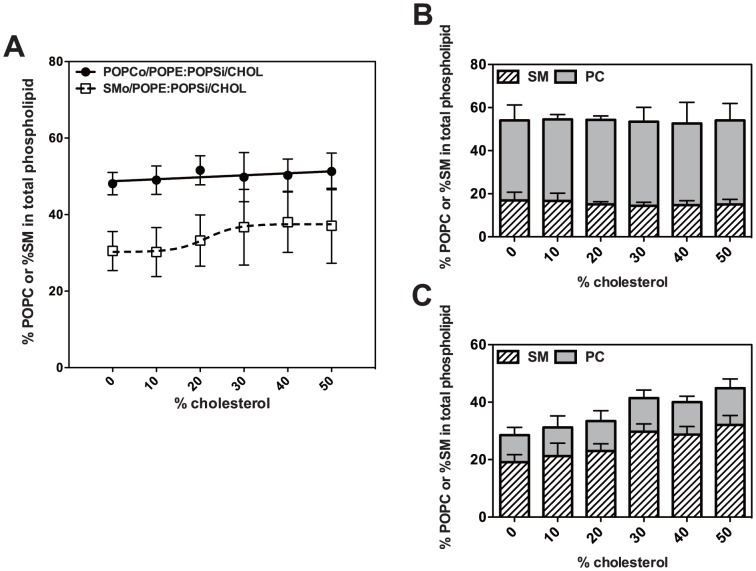
Exchange efficiency of donor lipid into exchange vesicles containing varies amount of cholesterol. Exchange vesicles were prepared using pure POPC or pure SM (A), 1∶1 SM/POPC (B), or 4∶1 SM/POPC (C) in the donor vesicles. Acceptor vesicles were composed of 1∶1 POPE/POPS with different amounts of cholesterol. % POPC or % SM of total phospholipid in exchange vesicles was determined by HP-TLC versus standard curves in which different amounts of each lipid was loaded on the HP-TLC plate. Average values (mean) and S.D., or range if n = 2, are shown. Sample numbers were n = 6 for POPCo/POPE:POPSi/cholesterol; n = 4 for SMo/POPE:POPSi/cholesterol and 1∶1 SM:POPCo/POPE:POPSi/cholesterol; and n = 2 for 4∶1 SM:POPC/POPE:POPSi/cholesterol.

We next examined the effect of varying outer leaflet lipid and cholesterol content upon the efficiency of exchange. First, exchange of POPC into 1∶1 POPE/POPS vesicles containing different amounts of cholesterol (0–50 mol%) was carried out. Based on HP-TLC (Fig. S5 in File S1), the POPC content as a % of total phospholipid in the exchange vesicles was ∼50% over the entire range (0–50 mol%) of cholesterol contents (Fig. 3A and Table S2 in File S1). The theoretical value for complete outer leaflet exchange for 140 nm diameter LUVs is about 53%, because the outer leaflet has 53% of the total surface area for this diameter. Thus, slightly over 90% of the lipids in the acceptor vesicle outer leaflet were exchanged (after correcting for contamination by donor POPC vesicles). As noted above, % cholesterol content is not affected by lipid exchange.

A lower efficiency of exchange with SM was observed in experiments in which pure SM was used as the donor lipid. In this case, exchange vesicles had a SM content (as a % of total phospholipid) of 30–40% (Fig. 3A and Table S2 in File S1). This is equivalent to only 55–73% exchange of vesicle outer leaflet lipid with SM. The level of exchange increased with increasing cholesterol. To imitate plasma membrane more precisely, asymmetric vesicles with an outer leaflet composed of a SM/POPC mixture would be desirable. Therefore, we investigated the efficiency of exchange of SM/POPC donor mixtures into POPE/POPS/cholesterol acceptor vesicles. Lipid exchange was found to be dependent both on the SM/POPC ratio and cholesterol content. The efficiency of SM/POPC exchange was close to or above 90% for all cholesterol concentrations from 0 to 50 mol% for samples in which the donor lipid was a mixture of 1∶1 SM/POPC (Fig. 3B and Table S2 in File S1), but increased from 60% to 82% as cholesterol increased from 0 to 50 mol% for samples in which the donor lipid was a mixture of 2∶1 SM/POPC, and increased from 50% to 82% as cholesterol increased from 0 to 50 mol% for samples in which the donor lipid was a mixture of 4∶1 SM/POPC (Fig. 3C and Table S2 in File S1). The SM/POPC ratio in the exchange vesicles increased as the SM/POPC ratio in the donor lipid mixture increased (Fig. 3 and Table S2 in File S1). However, the SM/POPC ratio in the exchange vesicles was lower than that in the donor lipid mixture, confirming that SM exchanges less efficiently than POPC. For all of these mixtures, the POPE:POPS ratio after exchange (ranging between 1∶1 to 1.5∶1) remains close to that for the original acceptor vesicles (1∶1), although some variability was seen (Table S2 in File S1).

These experiments show that vesicles with compositions that mimic that of mammalian membranes can be prepared by lipid exchange, although the efficiency of exchange is somewhat lipid dependent. It is important to note that there was a generally high (80–95%) replacement of outer leaflet lipids with POPC or mixtures of SM and POPC when physiological cholesterol concentrations were present (≥30 mol%).

### Measurement of lipid asymmetry after lipid exchange

By themselves, the studies above do not demonstrate that the exchange vesicles have an asymmetric lipid distribution. Previous studies have shown that lipid exchange catalyzed by MβCD only involves outer leaflet lipids, and results in asymmetric vesicle formation except when unusual lipids that can undergo fast transverse diffusion (flip-flop) are present [10], but this might not be the case for HPαCD-induced exchange or for the high cholesterol contents in some of the vesicles used in this study. Thus, it was important to show that when using HPαCD the exchange vesicles had an asymmetric lipid distribution.

**Figure 4:**
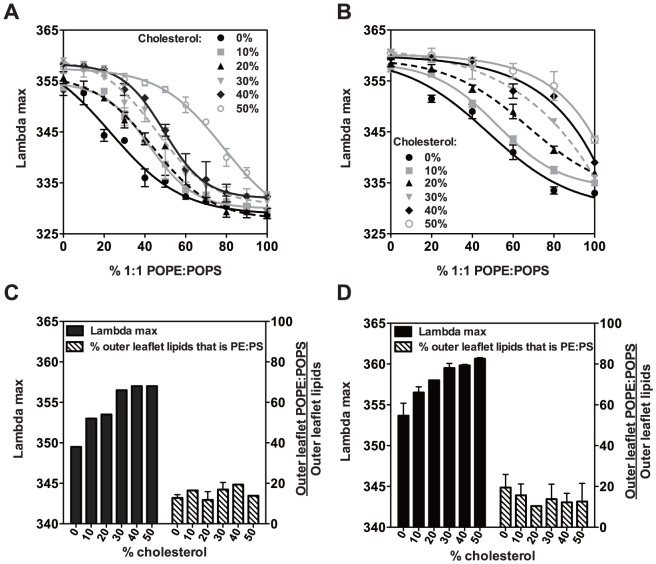
Assay of lipid asymmetry detected by binding of hydrophobic helix pL4A18. (A–B) Dependence of fluorescence emission λ_max_ of pL4A18 peptide upon the fraction of 1∶1 POPE/POPS in symmetric vesicles composed of 1∶1 POPE/POPS mixed with POPC (A), or 1∶1 SM/POPC (B), plus different amounts of cholesterol. % POPE:POPS equals sum of % POPE plus % POPS. Average values (mean) and S.D. from three samples are shown. (C–D) Fluorescence emission λ_max_ (black bar) of pL4A18 peptide binding to exchange (asymmetric) vesicles and calculated outer leaflet lipids that were POPE:POPS (striped bars) are shown for POPCo/POPE:POPSi/cholesterol (C), or 1∶1 SM:POPCo/POPE:POPSi/cholesterol (D). The % of outer leaflet lipids that were POPE:POPS was calculated from the standard curves (A–B) fitted to Boltzmann Sigmoid curves (GraphPad Prism software, La Jolla, CA). Average values (mean) and S.D. are shown with n = 4 for POPCo/POPE:POPSi/cholesterol and n = 3 for 1∶1 SM:POPCo/POPE:POPSi/cholesterol vesicles.

To detect the presence of anionic lipids on the outer leaflet, the binding of the cationic peptide pL4A18 (acetyl-K_2_LA_9_LWLA_9_LK_2_-amide) was used. pL4A18 is a hydrophobic helix with Lys residues that give it increased affinity for anionic membranes [19]. Its membrane-association can be monitored by the blue shift of its Trp fluorescence emission λ_max_. Because pL4A18 is added externally to vesicles, only the lipid composition of the outer leaflet should affect its binding. First, standard curves of emission λ_max_ of pL4A18 peptide mixed with symmetric vesicles vs. % (POPE:POPS) were generated (Fig. 4A,B). Increasing amounts of PE/PS in the vesicles progressively blue-shifted λ_max_. Because cholesterol was found to influence pL4A18 interaction with vesicles, standard curves were generated for each cholesterol concentration used.

Next, the λ_max_ of pL4A18 peptide binding to asymmetric vesicles was compared to the standard curves to estimate the percentage of POPE and POPS in the exchange vesicle outer leaflets. Fig. 4C shows that in POPCo/POPE:POPSi/cholesterol vesicles, ∼15–20% of phospholipids in the outer leaflet were PE/PS at all cholesterol concentrations between 0 and 50 mol%. This amount of PE/PS is just slightly more than expected from the observation that ∼90% of outer leaflet lipids are exchanged with PC, confirming a very high degree of asymmetry. Vesicles were also highly asymmetric when exchange was carried out with 1∶1 SM/POPC donor vesicles, in which PE/PS again composed about 15% of lipids in the outer leaflet (Fig. 4D). At least at high cholesterol levels, where exchange was efficient, a similar high level of asymmetry was observed when the donor lipids were 2∶1 or 4∶1 SM:POPC (Fig. S6 in File S1). As expected from the low efficiency of exchange, a high level of outer leaflet PE/PS was observed when exchange was carried out with SM as the only donor lipid (Fig. S6 in File S1).

**Figure 5:**
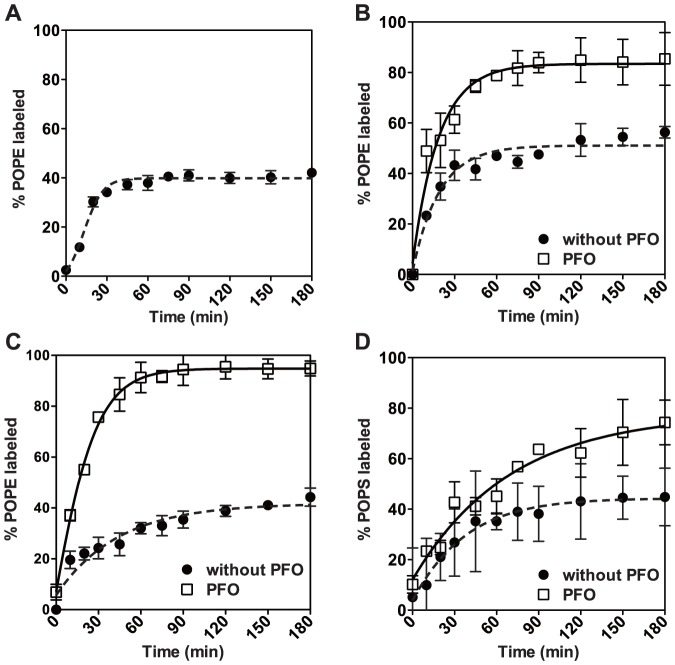
TNBS labeling of LUVs with or without Perfringolysin O (PFO). Samples contained 100 µl of (A) 1∶1 POPC/POPE, (B) 3∶3∶4 POPC/POPE/cholesterol or (C–D) 2∶2∶2∶4 POPC/POPE/POPS/cholesterol LUVs with or without ∼20 µg PFO and 900 µl, 1mM TNBS in NaHCO_3_ pH 9. Final lipid concentration was 200 µM. After TNBS addition, samples were incubated at room temperature for various times. % POPE labeled =  [1−(POPE/POPC)_before TNBS labeling_/(POPE/POPC)_after TNBS labeling_] ×100% (A–C) or % POPS labeled  =  [1−(POPS/POPC)_before TNBS labeling_/(POPS/POPC)_after TNBS labeling_] ×100% (D). POPE/POPC or POPS/POPC is the ratio of the amount of POPE or POPS to that of POPC calculated from HP-TLC of lipid extracts from vesicles. Average values and range from duplicates are shown.

Chemical labeling by trinitrobenzensulfonate (TNBS) was used to confirm the results obtained from peptide binding. TNBS covalently reacts with the amino groups of PE and PS. TNBS is relatively membrane-impermeant, and has been used to assess the TM topology of aminophospholipids [20], [21]. To investigate the utility of TNBS labeling for our vesicles, symmetric vesicles composed of 1∶1 POPC/POPE, 3∶3∶4 POPC/POPE/cholesterol or 2∶2∶2∶4 POPC/POPE/POPS/cholesterol were incubated with TNBS for various times. HP-TLC analysis of residual unlabeled POPE (which migrated at a position distinct from that of TNBS-labeled POPE) showed that PE labeling by 60 min is ∼42% in POPC/POPE (Fig. 5A), ∼50% in POPC/POPE/cholesterol (Fig. 5B), and ∼40% in POPC/POPE/POPS/cholesterol (Fig. 5C), close to, but slightly less than the expected 50–55% labeling if TNBS only labels outer leaflet PE. This could mean either that the reaction with outer leaflet lipids did not quite go to completion, or that, instead of being randomly located, PE located slightly more favorably in the inner leaflet. To evaluate the extent of reaction and confirm labeling only involved outer leaflet lipids, labeling was evaluated in the presence of the cholesterol-dependent, pore-forming protein perfringolysin O (PFO) [14]. In the presence of PFO, labeling increased to 80–95% of total PE (Fig. 5B,C), indicating that the TNBS concentration and incubation times used were sufficient to react with exposed POPE molecules. (Residual unreacted POPE reflected a lack of pore formation in some vesicles. When fluorescent dextrans were trapped in lipid vesicles with the compositions and conditions used in Fig. 5B, ∼40% was not released by PFO, indicating that 40% of inner leaflet POPE was inaccessible to TNBS. This predicts that labeling of total POPE should be ∼80%, as was observed.) Reaction of TNBS with POPS was slower and more incomplete than that with POPE (Fig. 5D), so TNBS was not used to evaluate POPS asymmetry.

**Figure 6:**
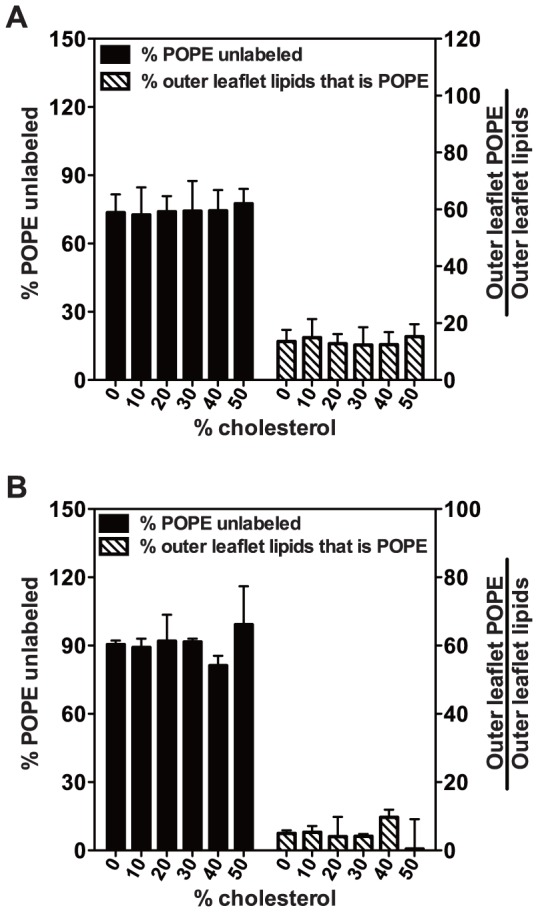
TNBS labeling of POPE in exchange (asymmetric) vesicles outer leaflet. (A) POPCo/POPE:POPSi/cholesterol and (B) 1∶1 SM:POPCo/POPE:POPSi/cholesterol. Labeling was for 60 min using the protocol in Fig. 5. Black bar shows the % of POPE unlabeled, which equals (POPE/POPC)_before TNBS labeling_/(POPE/POPC)_after TNBS labeling_×100%. The % of outer leaflet lipids that was POPE after exchange (striped bar)  =  (100% – % POPE unlabeled) × the fraction of vesicle lipids that was POPE (see Table S2 in File S1)/53%. This assumes ∼53% of LUV lipid is in the outer leaflet. Average values (mean) and S.D. are shown and n = 4 for POPCo/POPE:POPSi/cholesterol and n = 3 for 1∶1 SM:POPCo/POPE:POPSi/cholesterol vesicles.

TNBS labeling of POPCo/POPE:POPSi/cholesterol exchange vesicles showed ∼80% of total PE was protected from labeling (Fig. 6A). This level of labeling was not affected by cholesterol concentration. Assuming only outer leaflet PE was labeled, the level of labeling observed corresponds to the outer leaflet being composed of 10–15% PE.

In vesicles into which a mixture of SM and POPC was exchanged, and in which the overall level of lipid exchange was high, the level of PE asymmetry appeared to be a little higher than that with POPC exchange (with ∼5% of PE in the outer leaflet) (Fig. 6B). In cases in which HP-TLC showed the level of lipid exchange was low (Table S2), there was a high level of POPE labeling by TNBS, as expected (Fig. S7 in File S1).

With POPCo/POPE:POPSi vesicles and POPCo/POPE:POPSi/40 mol% cholesterol vesicles both peptide binding and TNBS labeling detected no increase in the amount of PE or PS in the outer leaflet for at least several days (Fig. S8 in File S1). This indicates that asymmetry is highly stable.

Overall, the values for the level of outer leaflet lipid exchange from HP-TLC, and those for POPE and POPS asymmetry from peptide binding and TNBS labeling, were in close agreement with each other, showing that the exchange vesicles were highly asymmetric. It should be noted that cholesterol itself should not have appreciable asymmetry in our vesicles, as it flips rapidly across membranes, and it should be close to randomly distributed in the inner and outer leaflet in the original acceptor vesicles (see Discussion).

## Discussion

### Preparation of asymmetric LUVs

MβCD-mediated phospholipid exchange has been used to produce asymmetric SUVs and LUVs in our previous studies [9], [11]. However, because MβCD has a high affinity for cholesterol, it has been difficult to prepare vesicles with an asymmetric phospholipid distribution and with cholesterol. We previously circumvented this by introducing cholesterol to preformed asymmetric vesicles in a second step using MβCD concentrations too low to affect phospholipid composition [9], but controlling the amount of cholesterol introduced and achieving high vesicle yields was difficult. To overcome these difficulties, we used HPαCD. HPαCD has a smaller hydrophobic cavity (diameter ∼5.7 Å) than MβCD (diameter ∼7.8 Å), which either greatly reduces or eliminates its affinity for cholesterol. This allowed us to develop a method in which cholesterol is embedded in acceptor vesicles prior to phospholipid exchange.

The HPαCD-induced lipid exchange method was introduced to make asymmetric LUVs with a wide range of cholesterol concentrations (0–50 mol%) and in which the outer leaflets are composed (largely) of SM or POPC or a mixture of SM and POPC while inner leaflets contain the aminophospholipids POPE and POPS. We demonstrated that the resulting vesicles are highly asymmetric and that asymmetry seems to be highly stable.

The amount of exchange observed is somewhat greater than that expected based on simple HPαCD induced equilibration of the outer leaflet lipids of donor and acceptor vesicles. This may reflect a difference in the affinity of HPαCD for different lipids, or that for some reason an asymmetric lipid distribution is lower energy than a symmetric one.

The exact distribution of cholesterol in the inner and outer leaflet is hard to define, but it should have been nearly equal in the inner leaflet prior to lipid exchange, and is likely to remain nearly equal in the two leaflets after lipid exchange due to the fact that cholesterol flips quickly between leaflets, and the requirement for lipid mass balance in the inner and outer leaflet, despite the fact that a higher affinity of cholesterol for outer leaflet lipid would favor an excess of cholesterol in the outer leaflet. Of course, if HPαCD catalyzed a net increase or decrease in total lipid in the acceptor vesicles, then cholesterol could flow to the leaflet with the lipid deficit, which would result in a more unequal cholesterol distribution. However, we do not observe a change in vesicle size after exchange. A change in size would be expected to accompany net lipid gain or loss.

It should be noted that use of HPαCD in place of MβCD may introduce some complications in asymmetric vesicle preparation. For example, exchange of SM by HPαCD is somewhat less efficient than by MβCD. HPαCD concentration, donor and acceptor vesicles concentrations, and centrifugation conditions (including sucrose concentrations needed) giving maximal lipid exchange and most facile exchange vesicle purification may all have to be carefully defined when designing asymmetric vesicles with new lipid compositions.

### Implications and future applications of asymmetric vesicles

We believe that the cholesterol-containing asymmetric vesicles introduced in this report are the model membrane vesicles that most closely resemble natural mammalian plasma membrane to date. Of course, it should be possible to prepare even more realistic membranes by using more complex lipid mixtures (e.g. natural lipids with a mixture of acyl chain compositions) in place of simple synthetic lipids. However, the vesicles prepared in this report incorporate the most essential features of plasma membrane both in terms of lipid composition and asymmetry, and thus we feel it is appropriate to call them “artificial plasma membrane mimicking” (“PMm”) vesicles. These vesicles can be formed using POPC in the outer leaflet in order to imitate liquid disordered regions of the plasma membrane, or with both SM and POPC in the outer leaflet to imitate plasma membrane with co-existing liquid ordered and disordered domains [22], [23]. The latter vesicles should be especially useful to study the physical properties of lipid compositions that closely resemble plasma membrane, including such properties as membrane domain formation and coupling between the physical properties of the inner and outer lipid leaflets [24]. Asymmetric vesicles should also be especially useful for a more detailed examination of lipid-protein interactions than heretofore possible. Membrane proteins could be reconstituted into symmetric lipid vesicles by standard protocols, and then asymmetry introduced by lipid exchange. Such studies will require that lipid asymmetry be maintained in the presence of membrane-inserted protein. This may not be problematic, as prior studies have shown very hydrophobic TM helices do not destroy lipid asymmetry [9], and we have recently found that lipid asymmetry is maintained in the presence of membrane-inserted perfringolysin O [25].

## Supporting Information

File S1 **Figure S1, Lack of cholesterol extraction by HPαCD. Figure S2, HPαCD-induced exchange efficiency is dependent on the ratio of donor/acceptor vesicle concentration. Figure S3, Comparison of vesicle size before and after HPαCD-induced lipid exchange. Figure S4, Schematic representation of protocol for measuring contamination of asymmetric vesicles by donor vesicles using C_6_-NBD-PC. Figure S5, HP-TLC chromatograms of asymmetric vesicle preparations. Figure S6, Assay of lipid asymmetry detected by binding of hydrophobic helix pL4A18. Figure S7, TNBS labeling of POPE in exchange (asymmetric) vesicles outer leaflet. Figure S8, Stability of exchange (asymmetric) vesicles. Table S1, Measurement of contamination from “donor” vesicles by NBD reduction. Table S2, Lipid composition in exchange vesicles.**
(DOCX)
