# Supplementary material for: Preparation of Artificial Plasma Membrane Mimicking Vesicles with Lipid Asymmetry
Source: PLoS One. 2014 Jan 28;9(1):e87903. doi: 10.1371/journal.pone.0087903 (PMC3905041; doi:10.1371/journal.pone.0087903)
Supplement: File S1 — Figure S1, Lack of cholesterol extraction by HPαCD. Figure S2, HPαCD-induced exchange efficiency is dependent on the ratio of donor/acceptor vesicle concentration. Figure S3, Comparison of vesicle size before and after HPαCD-induced lipid exchange. Figure S4, Schematic representation of protocol for measuring contamination of asymmetric vesicles by donor vesicles using C6-NBD-PC. Figure S5, HP-TLC chromatograms of asymmetric vesicle preparations. Figure S6, Assay of lipid asymmetry detected by binding of hydrophobic helix pL4A18. Figure S7, TNBS labeling of POPE in exchange (asymmetric) vesicles outer leaflet. Figure S8, Stability of exchange (asymmetric) vesicles. Table S1, Measurement of contamination from “donor” vesicles by NBD reduction. Table S2, Lipid composition in exchange vesicles. (DOCX) [file pone.0087903.s001.docx]

**File S1. Supporting Information for *“Preparation of Artificial Plasma Membrane Mimicking Vesicles with Lipid Asymmetry”* by Qingqing Lin and Erwin London**

**Table S1. Measurement of contamination from “donor” vesicles by NBD reduction**

| **Sample** | **NBD fluorescence** | **POPC amount (µmol)** | **NBD F/**  **µmol PC** | **% of lipid in pellet coming from donor vesicle contamination** |
| --- | --- | --- | --- | --- |
| **Before centrifugation** | 266 |  |  |  |
| **1^st^ supernatant** | 283 | 4.32 | 65 |  |
| **2^nd^ supernatant** | 2.83 | ~ 0 |  |  |
| **Final pellet** | 0.55 | 0.32 | 1.7 | 2.57 |

Donor vesicles composed of POPC MLV were prepared with 0.1 mol% C_6_-NBD-PC. Acceptor vesicles contained POPE/POPS with 40mol% cholesterol. The amount of POPC in the supernatant and pellet from each round of centrifugation was measured by HP-TLC, the amount of NBD lipids was determined from its fluorescence. Total recovery of POPC in the supernatants and pellet was about 58%. POPC in the second supernatant was below detection limits. NBD fluorescence shown is after subtraction of background derived from a preparation lacking C_6_-NBD-PC.

**Table S2. Lipid composition in exchange vesicles**

| **Exchange vesicles** | **% CHOL in total lipids** | **% SM**  **in total phospholipid** | **% POPC**  **in total phospholipid** | **% POPE**  **in total phospholipid** | **% POPS**  **in total phospholipid** |
| --- | --- | --- | --- | --- | --- |
| **POPCo/** | 0 | na | 48.1 ± 2.9 | 27.2 ± 3.1 | 24.7 ± 3.3 |
| **1:1 POPE:POPSi/CHOL** | 10 | na | 49.0 ± 3.7 | 28.8 ± 7.5 | 22.2 ± 5.0 |
|  | 20 | na | 51.6 ± 3.8 | 26.1 ± 7.1 | 22.3 ± 4.1 |
|  | 30 | na | 49.8 ± 6.4 | 25.3 ± 1.6 | 24.9 ± 7.0 |
|  | 40 | na | 50.3 ± 4.2 | 25.7 ± 4.3 | 24.0 ± 6.9 |
|  | 50 | na | 51.3 ± 4.8 | 36.0 ± 7.1 | 22.7 ± 5.3 |
| **1:1 SM:POPCo/** | 0 | 16.9 ± 3.8 | 37.1 ± 7.2 | 27.5 ± 4.3 | 18.5 ± 5.3 |
| **1:1 POPE:POPSi/CHOL** | 10 | 16.6 ± 3.6 | 37.9 ± 2.3 | 26.0 ± 8.5 | 19.5 ± 3.9 |
|  | 20 | 15.1 ± 1.2 | 39.2 ± 1.8 | 26.4 ± 7.4 | 19.3 ± 4.9 |
|  | 30 | 14.4 ± 1.6 | 39.0 ± 6.7 | 26.1 ± 3.7 | 20.5 ± 1.6 |
|  | 40 | 14.7 ± 2.1 | 37.9 ± 9.8 | 27.3 ± 5.7 | 20.1 ± 3.5 |
|  | 50 | 15.0 ± 2.4 | 39.0 ± 7.9 | 27.4 ± 4.2 | 18.6 ± 2.1 |
| **2:1 SM:POPCo/** | 0 | 14.9 ± 0.1 | 19.1 ± 0.1 | 39.4 ± 0.2 | 26.6 ± 0.2 |
| **1:1 POPE:POPSi/CHOL** | 10 | 14.5 ± 1.8 | 20.5 ± 1.6 | 37.5 ± 0.1 | 27.5 ± 0.3 |
|  | 20 | 17.2 ± 0.1 | 20.3 ± 0.4 | 31.5 ± 2.4 | 31.1 ± 2.1 |
|  | 30 | 19.3 ± 1.2 | 22.9 ± 1.1 | 34.0 ± 1.6 | 23.8 ± 0.7 |
|  | 40 | 21.1 ± 0.6 | 22.5 ± 4.7 | 28.8 ± 5.2 | 27.6 ± 0.2 |
|  | 50 | 21.7 ± 0.4 | 23.4 ± 1.6 | 28.6 ± 3.7 | 26.3 ± 1.6 |
| **4:1 SM:POPCo/** | 0 | 19.1 ± 2.6 | 9.4 ± 2.7 | 41.9 ± 3.1 | 29.5 ± 3.3 |
| **1:1 POPE:POPSi/CHOL** | 10 | 21.2 ± 4.5 | 10.0 ± 4.0 | 37.1 ± 1.3 | 31.7 ± 7.1 |
|  | 20 | 23.0 ± 2.5 | 10.4 ± 3.6 | 35.4 ± 1.9 | 31.2 ± 4.1 |
|  | 30 | 29.7 ± 2.7 | 11.7 ± 2.8 | 29.4 ± 9.9 | 29.2 ± 9.1 |
|  | 40 | 28.7 ± 2.8 | 11.3 ± 2.1 | 31.1 ± 1.6 | 28.8 ± 6.6 |
|  | 50 | 32.1 ± 3.3 | 12.8 ± 3.2 | 27.9 ± 1.5 | 27.2 ± 8.1 |
| **SMo/** | 0 | 30.5 ± 5.1 | na | 42.2 ± 8.4 | 27.3 ± 9.6 |
| **1:1 POPE:POPSi/CHOL** | 10 | 30.2 ± 6.4 | na | 41.3 ± 9.3 | 28.5 ± 8.4 |
|  | 20 | 33.2 ± 6.7 | na | 38.7 ± 1.4 | 28.1 ± 9.4 |
|  | 30 | 36.7 ± 9.9 | na | 37.5 ± 5.7 | 25.8 ± 7.9 |
|  | 40 | 38.0 ± 7.9 | na | 37.4 ± 8.8 | 24.6 ± 5.7 |
|  | 50 | 37.1 ± 9.8 | na | 34.3 ± 8.6 | 28.6 ± 8.4 |

Exchange vesicles were prepared using POPC, or SM, or different ratios of SM/POPC in the donor vesicles. Acceptor vesicles were composed of 1:1 POPE/POPS with different amounts of cholesterol. % of each lipid in exchange vesicles was determined by HP-TLC versus standard curves in which different amounts of each lipid was loaded on the HP-TLC plate. Average (mean) and S.D. or range of duplicates is shown. Sample numbers were n=6 for POPCo/POPE:POPSi/cholesterol; n=4 for SMo/POPE:POPSi/cholesterol and 1:1 SM:POPCo/POPE:POPSi/cholesterol; and n=2 for 2:1 SM:POPCo/POPE:POPSi/cholesterol and 4:1 SM:POPCo/POPE:POPSi/cholesterol. Abbreviations: CHOL= cholesterol; na = not applicable.

**Figure S1. Lack of cholesterol extraction by HPαCD.** (A) Lack of [^3^H]-cholesterol extraction from symmetric LUVs by HPαCD. 1mM, 1ml 6:4 POPC/cholesterol LUVs with 0.2 mol% LW peptide and 2.5 µCi ^3^H-cholesterol were incubated with 16.8 mM HPαCD at 55 °C for 30 min. After ultracentrifugation, the fluorescence of LW peptide, which is non-exchangeable and gives the amount of vesicles (black bar), and [^3^H]-cholesterol radioactivity (gray bar) were measured in both supernatant and pellet. (B) Lack of extraction of [^3^H]-cholesterol by HPαCD during lipid exchange. 8 mM, 500 µl 3:3:4 POPE/POPS/cholesterol LUVs with 0.2 mol% LW peptide and 10 µCi [^3^H]-cholesterol were incubated with 16 mM, 500 µl POPC-HPαCD mixtures. After two rounds of ultracentrifugation, the LW peptide fluorescence (black bar) and [^3^H]-cholesterol radioactivity (gray bar) in the two supernatants and final pellet were measured. The fraction of the total recovered [^3^H]-cholesterol and recovered LW peptide is shown. The equal recoveries of vesicles and cholesterol indicate a lack of cholesterol extraction by HPαCD. Notice that the recovery of the exchange vesicles in the pellet is ~ 25%. This is a typical recovery. (C) Repeat of experiments in part B with various cholesterol concentrations. Ratios of the fraction of [^3^H]-cholesterol radioactivity to the fraction of LW peptide fluorescence in the final pellet is shown. A value of one indicates a lack of cholesterol extraction. Average of duplicates and range is shown.

**Figure S2. HPαCD-induced exchange efficiency is dependent on the ratio of donor/acceptor vesicle concentration.** The “acceptor” vesicle stock solution was composed of 500 μl, 4 mM, 8mM or 12 mM 1:1 POPE/POPS or 3:3:4 POPE/POPS/cholesterol with 25% (w/v) sucrose trapped inside. The “donor” vesicle stock solution was composed of 600 μl 13.3 mM POPC or SM with 105 mM HPαCD, which was then incubated overnight at 55 ^o^C. After mixing of donor and acceptor solutions, incubation at 55 °C for 30 min and centrifugation, the % POPC or %SM in exchange vesicles was determined by band intensity on HP-TLC versus standard curves in which different amounts of each lipids was loaded on the HP-TLC plate.

**Figure S3. Comparison of vesicle size before and after HPαCD-induced lipid exchange.** 1:1 POPE/POPS LUVs containing different mol% cholesterol were prepared by extrusion through a 100 nm-pore size polycarbonate filter. LUV vesicle size before (black bar) and after (gray bar) lipid exchange using POPC as donor and purification of the resulting asymmetric vesicles, was determined by dynamic light scattering.

**Figure S4.** **Schematic representation of protocol for measuring contamination of asymmetric vesicles by donor vesicles using C_6_-NBD-PC.** Notice that after dithionite reduction any NBD lipids in the pellet must be due to donor vesicle contamination.


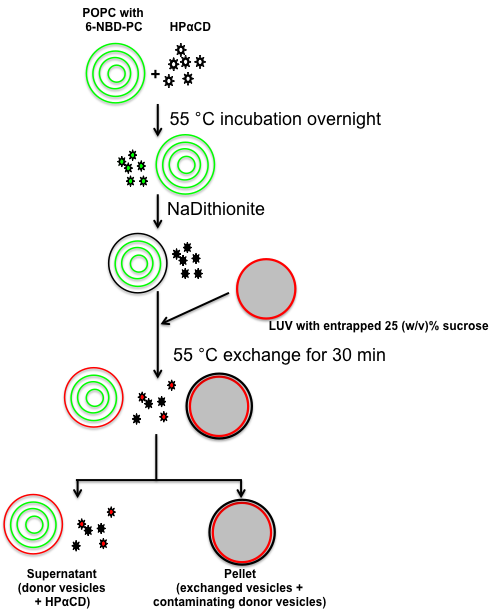


**Figure S5. HP-TLC chromatograms of asymmetric vesicle preparations.** (A) POPCo/POPE:POPSi/cholesterol; (B) SMo/POPE:POPSi/cholesterol; and (C) 1:1 SM:POPCo/POPE:POPSi/cholesterol. 200 µl of resuspended asymmetric vesicles (~0.2 µmol total lipid) in PBS pH 7.4 were extracted using 2:2:1 (v/v) chloroform/methanol/(vesicle in PBS). The dried and redissolved extracted lipids were then applied to HP-TLC. Note that SM stains more darkly than an equal amount of POPC.

**Figure S6.** **Assay of lipid asymmetry detected by binding of hydrophobic helix pL4A18.** (A) Dependence of fluorescence emission λ_max_ of pL4A18 peptide upon the fraction of 1:1 POPE/POPS in symmetric vesicles composed of SM with different amount of cholesterol. % POPE:POPS equals sum of % POPE plus % POPS. Average values (mean) and S.D. from three samples are shown. (B-D) Fluorescence emission λ_max_ (black bar) of pL4A18 peptide to exchange (asymmetric) vesicles and calculated outer leaflet lipids that were POPE:POPS (striped bars) for SMo/POPE:POPSi/cholesterol (B), 2:1 SM:POPCo/POPE:POPSi/cholesterol (C), or 4:1 SM:POPCo/POPE:POPSi/cholesterol (D). The % of outer leaflet lipids that were POPE:POPS was calculated from the standard curves (Fig. 4B for samples with SM and POPC) fitted to Boltzmann Sigmoid curves (GraphPad Prism software, La Jolla, CA). Average values (mean) and S.D. are shown and n=4 for SMo/POPE:POPSi/CHOL vesicles. Single values are shown for 2:1 SM:POPCo/POPE:POPSi/cholesterol and 4:1 SM:POPCo/POPE:POPSi/cholesterol vesicles.

**Figure S7.** **TNBS labeling of POPE in exchange (asymmetric) vesicles outer leaflet.** (A) SMo/POPE:POPSi/cholesterol; (B) 2:1 SM:POPCo/POPE:POPSi/cholesterol and (C) 4:1 SM:POPCo/POPE:POPSi/cholesterol. Labeling was for 60 min using the protocol in Fig. 5. Black bar shows the % POPE unlabeled, which equals (POPE/SM)_before TNBS labeling_/(POPE/SM)_after TNBS labeling_ x 100% in SMo/POPE:POPSi/cholesterol vesicles or equals (POPE/POPC)_before TNBS labeling_/(POPE/POPC)_after TNBS labeling_ x 100% in POPC-containing vesicles. The % of outer leaflet lipids that was POPE (striped bar) = (100% - % POPE unlabeled) × fraction of vesicle lipids that was POPE (see Table S2)/53%. This assumes ~ 53% of LUV lipid is in the outer leaflet. Average of triplicates and S.D. are shown for SMo/POPE:POPSi/cholesterol. Average of duplicates and range are shown for 2:1 SM:POPCo/POPE:POPSi/cholesterol and 4:1 SM:POPCo/POPE:POPSi/cholesterol vesicles.

**Figure S8. Stability of exchange (asymmetric) vesicles.** (A) Fluorescence emission λ_max_ of pL4A18 peptide added to POPCo/POPE:POPSi (●) and POPCo/POPE:POPSi/40 mol% cholesterol (□) exchange vesicles. (B) TNBS labeling of POPE in POPCo/POPE:POPSi (●) and POPCo/POPE:POPSi/40 mol% cholesterol (□) exchange vesicles. Peptide was added or TNBS labeling carried out after incubation of vesicles at room temperature for the time shown on the x-axis.
